# Aluminum/Stainless Steel Clad Materials Fabricated via Spark Plasma Sintering

**DOI:** 10.3390/ma13010239

**Published:** 2020-01-06

**Authors:** Kwangjae Park, Dasom Kim, Kyungju Kim, Hansang Kwon

**Affiliations:** 1 Department of Materials System Engineering, Pukyong National University, 365 Sinseon-ro, Nam-gu, Busan 48547, Korea; park.kwangjae@j.mbox.nagoya-u.ac.jp (K.P.); dasom.kim@f.mbox.nagoya-u.ac.jp (D.K.); 2 The Industrial Science Technology Research Center, Pukyong National University, 365 Sinseon-ro, Nam-gu, Busan 48547, Korea; ngm13@ngm.re.kr; 3 Department of R&D, Next Generation Materials Co., Ltd., 365 Sinseon-ro, Nam-gu, Busan 48547, Korea

**Keywords:** aluminum, stainless steel, clad materials, intermetallic compounds, spark plasma sintering

## Abstract

Aluminum (Al)/stainless steel (SUS) clad materials were fabricated via the process of spark plasma sintering (SPS) using Al powder/bulk and an SUS sheet. Three Al/SUS clad types were fabricated: powder/bulk (P/B), bulk/bulk (B/B), and bulk/powder/bulk (B/P/B). During the SPS, Al and SUS reacted with each other, and intermetallic compounds were created in the clads. The thermal conductivity and thermal-expansion coefficient were measured using a laser flash analyzer and dynamic mechanical analyzer, respectively. The Al/SUS (P/B) clad had a thermal conductivity of 159.5 W/mK and coefficient of thermal expansion of 15.3 × 10^−6^/°C. To analyze the mechanical properties, Vickers hardness and three-point bending tests were conducted. The Al/SUS (P/B) clad had a flexural strength of about 204 MPa. The Al/SUS clads fabricated via SPS in this study are suitable for use in applications in various engineering fields requiring materials with high heat dissipation and high heat resistance.

## 1. Introduction

The demand for high-performance materials in various industrial fields is increasing, and the material industry is gaining importance as a technology-intensive and high-value industry [1,2,3,4]. Metallic materials are core base materials accounting for more than 60% of all basic industrial materials. They are widely used not only in infrastructure (bridges, harbors, architecture, power generation, etc.) and basic industries (chemicals, machinery, automobiles, shipbuilding, etc.), but also as a core base material in high-technology industries, such as robotics and information technology. Currently, the need for advanced functional materials to address environmental and energy problems is increasing in various global industries in response to energy-reduction policies and CO_2_ emission regulations [5,6,7].

Aluminum (Al) and stainless steel (SUS) are typical metal materials used in many industrial fields. Al is a typical lightweight metal with a density as low as one-third that of steel, and it is used as a functional material in next-generation automobiles and transportation equipment [8]. Stainless steel has excellent corrosion resistance and mechanical strength, and is hence used in various applications, such as industrial materials, automotive and aerospace structures, construction materials, and cooking utensils [9]. However, metal-matrix composites that combine the individual performances of metal materials can create high-performance functional materials that surpass the limited function of single-metal materials. Therefore, metal-matrix composites are highly valued in industrial applications because they can exhibit various functions by appropriately utilizing the functionality of each original material [10]. There are various methods for fabricating metal-matrix composites; the clad material fabricated via the process of cladding is a laminated metal composite with a structure in which dissimilar metal materials of different physical properties are bonded, and its function can be easily controlled depending on the kind and content of the metals to be bonded [11,12,13].

Recently, Yilamu et al. attempted to fabricate pure Al (A1100) and SUS (430SS) clad materials via hot rolling at 673 K [14]. They reported that the bending characteristics were significantly influenced by the sheet thicknesses and sheet-set conditions of Al and SUS. Liu et al. investigated clads fabricated from a 2 mm thick SUS (1Cr18Ni9Ti) and Al alloy (AlSi7Mg) via the semisolid joining technique [15]. The researchers achieved a maximal interfacial shear strength of about 106.17 MPa when the solid fraction of the Al alloy was 30%. Akramifard et al. investigated Al/304L/Al clad sheets stacked with Al alloy and SUS (304L) via cold-roll bonding [16]. Guo et al. reported the fabrication of not only clad sheets but also clad pipes [17]. The bimetal pipes comprising Al and SUS (316L) pipes were fabricated via the method of explosive cladding.

In this study, we manufactured Al/SUS clad materials via spark plasma sintering (SPS) on the basis of the powder process for the easy control of interfacial properties between the different components of the clad materials. When compared with the conventional process of rolling, SPS is advantageous to the manufacture of clad materials with various interface properties. This is achieved by controlling the physical properties of the bonding interface by controlling the shape and size of the powder. In addition, the process of SPS can minimize the generation of impurities at the interfaces between the different materials owing to rapid heating and high-speed sintering. The Al/SUS clad materials in this study were fabricated from Al powder (or sheet) and SUS sheet via SPS. The microstructures and values of Vickers hardness, flexural strength, thermal conductivity, and thermal-expansion coefficient of the Al/SUS clad materials fabricated were analyzed.

## 2. Materials and Methods

### 2.1. Al/SUS Clad Material Fabrication 

**Figure 1:**
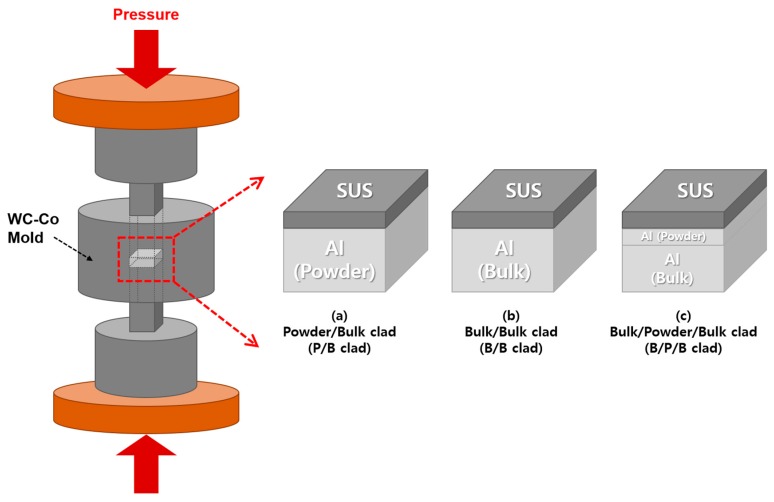
Schematic diagram of spark plasma sintering (SPS) and 3 kinds of aluminum/stainless steel (Al/SUS) clads.

First, we used pure Al powder (AlCO Engineering Co. Ltd., purity of 99.7%, particle size below 75 μm, Seosan, Korea) and sheet with a thickness of 1 mm (HAJIN Metal, Busan, Korea), and an SUS sheet with a thickness of 0.1 mm manufactured in-house with 30% Cr as the raw materials. The surface of the Al and SUS sheets was polished using 400 grit sandpaper to remove the oxide film. Three types of Al/SUS clad material samples were fabricated, as shown in Figure 1: (a) Al/SUS (powder/bulk, P/B) clad; (b) Al/SUS (bulk/bulk, B/B) clad; and (c) Al/Al/SUS (bulk/powder/bulk, B/P/B) clad. The Al powder or sheet and SUS sheet were stacked in a tungsten carbide-cobalt (WC-Co) mold (10 mm in width and 5 mm in depth). In particular, to fabricate samples for the flexure test, a WC-Co mold, 30 mm in width and 5 mm in depth, was used. Boron nitride (BN) was sprayed on the wall of the WC-Co mold and the carbon sheet above and below the WC-Co punch to prevent any reaction between the raw materials and mold. The Al/SUS clads were spark-plasma-sintered at 600 °C and 200 MPa in vacuum (about 1.6 Pa). During SPS, the temperature was increased from 25 to 600 °C at a rate of 30 °C/min and maintained at 600 °C for 10 min. The surface of the fabricated samples was polished using 400, 800, 1200, and 2000 grit sandpaper, in that order. The total thickness of the prepared Al/SUS clad samples was about 1 mm.

### 2.2. Al/SUS Clad Material Characterization

The densities of the Al/SUS clads were measured via the Archimedes method using analytical balance (ABJ 120-4M, Kern, Balingen, Germany). The microstructures were analyzed using a field emission scanning electron microscope (TESCAN, MIRA 3 LMH In-Beam, Brno, Czech Republic) and an energy dispersive X-ray spectroscopy (EDS) instrument (HORIBA, EX-400, Tokyo, Japan). The thermal conductivity of the Al/SUS clads was analyzed using a laser flash analyzer (LFA) (NETZSCH, LFA-467, Selb, Germany). The thermal-expansion coefficients were analyzed using a dynamic mechanical analyzer (PERKIN ELMER, DMA-7e, Waltham, MA, USA), and measurement direction was perpendicular to the interface. In addition, three-point-bending and Vickers hardness tests (Mitutoyo Corporation, HM-101, Kawasaki, Japan) were conducted to analyze the mechanical properties of the Al/SUS clads. The dimensions of the three-point-bending test specimens were 30 mm in width, 5 mm in depth, and 1 mm in height. The three-point bending test was conducted using a universal testing machine (INSTRON, INSTRON 5882, Norwood, MA, USA) with a cross head speed of 1 mm/min. The distance between supports was 25 mm. The Vickers hardness of each layer in the samples was measured 5 times according to Japanese Industrial Standard B 7725 and International Organization for Standardization (ISO) 6507-2, using a load of 0.3 kg for 5 s.

## 3. Results and Discussion

**Figure 2:**
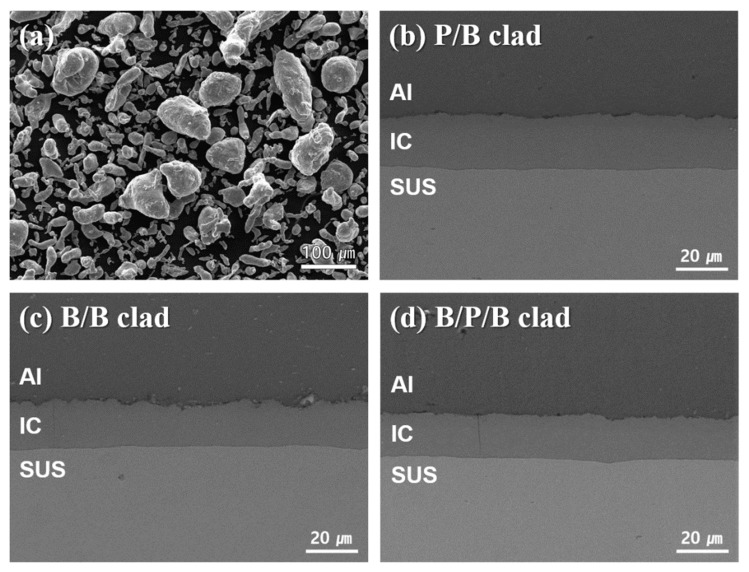
SEM micrographs of (**a**) raw Al powder, (**b**) powder/bulk (P/B), (**c**) bulk/bulk (B/B), and (**d**) bulk/powder/bulk (B/P/B) clads. IC, intermetallic compounds.

**Figure 3:**
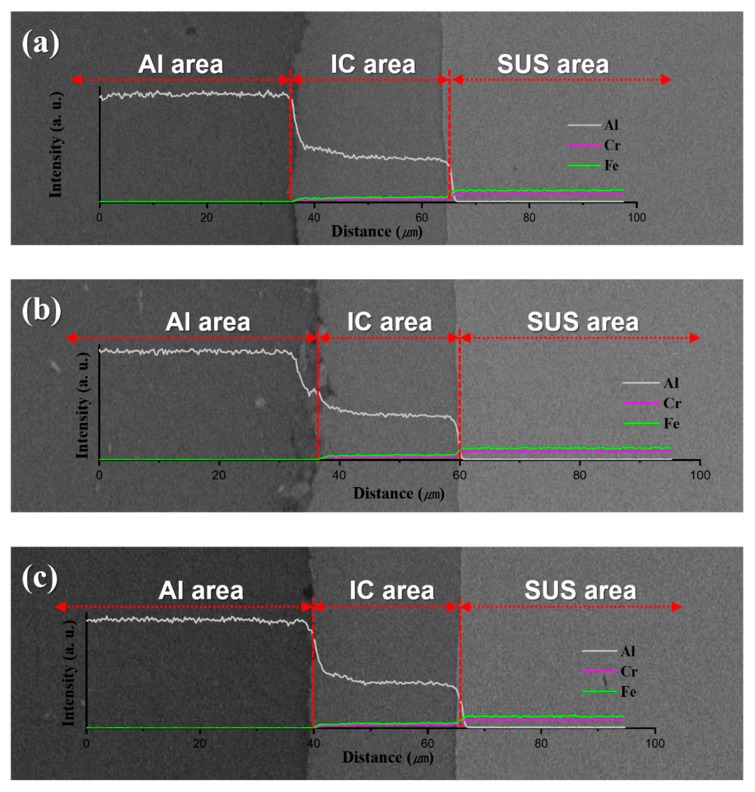
Energy dispersive X-ray spectroscopy (EDS) line scans of Al/SUS: (**a**) P/B, (**b**) B/B, and (**c**) B/P/B clads.

Figure 2 shows the morphology of the Al powder and the microstructures of the Al/SUS clads. As seen in Figure 2a, the Al powder presented irregular morphologies with several size distributions. The microstructures of the Al/SUS clads are shown in Figure 2b–d. All three Al/SUS clad types exhibited similar microstructures despite the differences between their Al forms (powder or bulk). In particular, intermetallic compounds (ICs) were produced between the Al and SUS phases, regardless of whether Al powder or bulk was used. It was assumed that the ICs were created by the reaction between the Al and SUS. To further clarify IC generation, the Al/SUS clads were analyzed via EDS line scan, as shown in Figure 3 (a: P/B clad, b: B/B clad, and c: B/P/B clad). Al, Fe, and Cr components were detected in the area of the ICs, which further validated that the ICs were formed from the reaction between Al and SUS. In our previous research [18,19], we fabricated Al-SUS316L composites using Al and SUS316L as the raw powders. Intermetallic compounds, such as Al_13_Fe_4_ and AlFe_3_, were created in the Al-SUS316L composites from the reaction between Al and SUS316L powder during SPS. In other words, it could be seen that the formation of ICs occurred during this research. This presented a two-dimensional cross-sectional situation that is not observed in the case of uniformly mixed composites using powders. Moreover, it was confirmed that the ICs had a uniform thickness of about 20 μm in the present study. This implied that Al and SUS reacted uniformly at the two contact surfaces via the microspark plasma mechanism [20] during SPS to produce ICs, suggesting the possibility of sinter bonding between the two different materials in the process of SPS. In addition, in our previous work [18], we confirmed that IC thickness was affected by the sintering temperature of the SPS process. When sintering temperature increased and approached the melting temperature of Al, some Al likely melted and infiltrated the SUS matrix, thereby resulting in increased IC creation [18]. Although Al powder and sheet were used in this work, it was assumed that IC thickness was similar because the sintering temperature was the same.

Table 1 presents the sintering densities and thermal properties of the Al/SUS clads. The relative densities of the three types of Al/SUS clads were greater than approximately 95%. As shown in Figure 2 and Figure 3, all clads were sintered in a form with almost no pores at the interface between Al and SUS, which resulted in their relatively high sintering densities. In general, it is well known that there are some difficulties in jointing Al and SUS [21,22,23]. The drastic difference between the melting points of the two materials causes Al combustion that could generate impurities and deteriorate material properties. An unfavorable bonding interface may be created owing to the difference between the thermal-expansion coefficients of the two materials. However, using the SPS method in this study, sintering was performed in a short period of time at a relatively low temperature and high pressure. Hence, clad materials with excellent bonding interfaces were produced.

**Figure 4:**
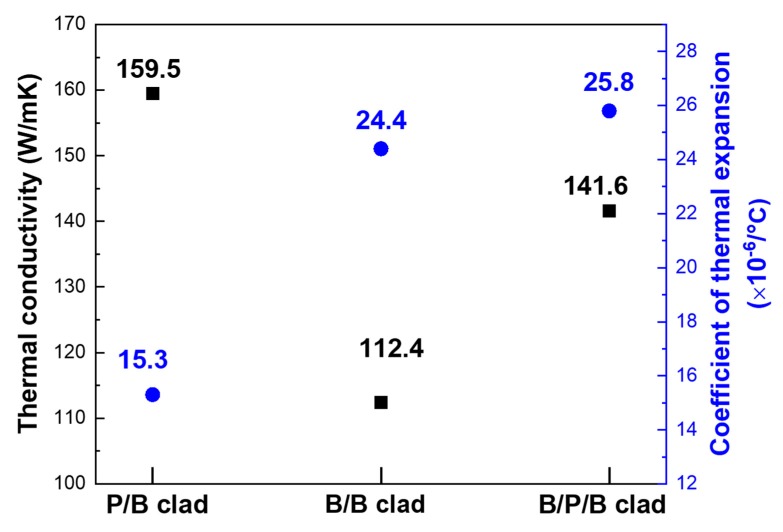
Thermal conductivities and coefficients of thermal expansion of Al/SUS clads.

The thermal-conductivity values of the Al/SUS clads, as analyzed via LFA, are presented in Figure 4. The Al/SUS (P/B) clad exhibited the highest value of thermal conductivity at 159.5 W/mK. The P/B and B/P/B clads had more grain boundaries than the B/B clad, which likely increased its thermal resistance owing to the large surface area of the P/B and B/P/B conditions. Nevertheless, these two clads had excellent thermal conductivity compared to the B/B clad because the interface between Al powder and SUS bulk formed an efficient heat-transfer interface rather than one of heat resistance. This interfacial structure was attributed to the formation of a favorable bond, which enabled efficient heat transfer between the two phases. However, the B/P/B clad had more grain boundaries than the P/B clad because it had two interfaces, which were SUS (Bulk)/Al (Powder) and Al (Powder)/Al (Bulk). For the B/P/B clad, more grain boundaries created greater thermal resistance rather than a heat-transfer interface when compared to P/B clad. We assumed that this was the reason P/B clad had higher value of thermal conductivity than that of the B/P/B clad.

The coefficients of thermal expansion of the Al/SUS clads are presented in Figure 4. The Al/SUS (P/B) clad had a value of 15.3 × 10^−6^/°C, the lowest between the three kinds of Al/SUS clads. The low coefficient of thermal expansion indicated high resistance to heat, such that the clad could withstand the mechanical stresses caused by shrinkage and expansion. In other words, we assumed that the Al/SUS (P/B) clad had outstanding heat resistance. On the basis of the thermal-property results, we inferred that the Al/SUS (P/B) clad could be applied as material exhibiting high heat dissipation and high heat resistance. However, further analysis is required to determine the mechanism by which the Al/SUS (P/B) clad gains its outstanding thermal properties, and this will be conducted in our future work.

**Figure 5:**
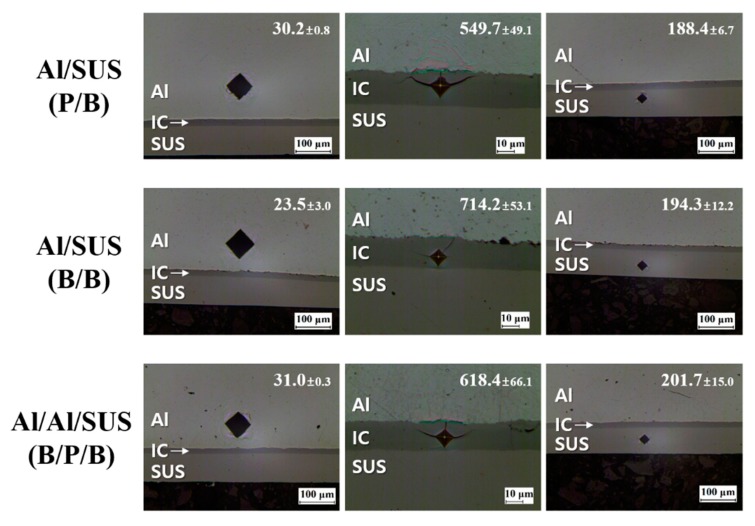
Vickers hardness values of Al, IC, and SUS areas in Al/SUS clads (inset average values and standard deviations of Vickers hardness).

To determine the mechanical properties of the clads, the Vickers hardness test was conducted as shown in Figure 5. The Al and SUS layers presented the typical Vickers hardness values of Al and SUS, respectively [24,25]. On the other hand, it was confirmed that the IC layers had very high values of Vickers hardness of about 549–714 HV. In addition, the Al–Fe ICs had high Vickers hardness values: 892 ± 6 HV, FeAl_2_; 903 ± 7 HV, Fe_2_Al_5_; and 691 ± 5 HV, FeAl_3_ [26]. In other words, these microhardness measurements presented the possibility of IC creation by reaction between Al and SUS.

**Figure 6:**
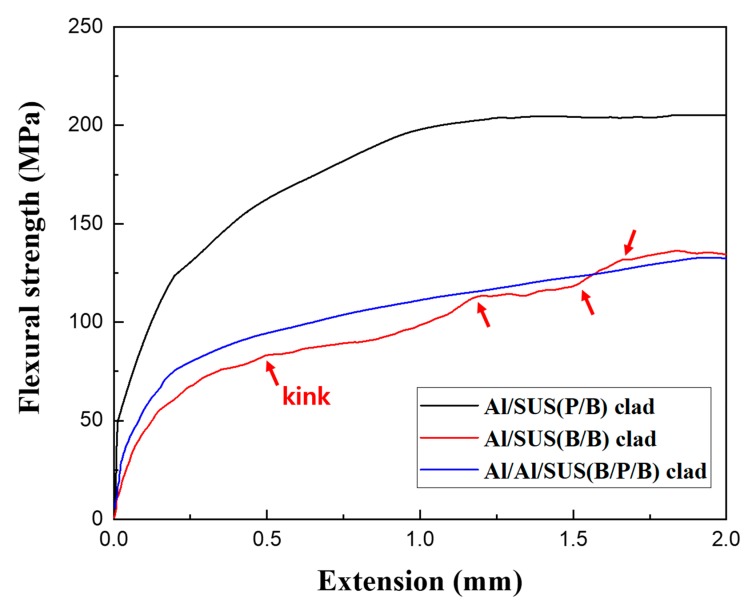
Flexural strength curves of Al/SUS clads determined via three-point bending test.

**Figure 7:**
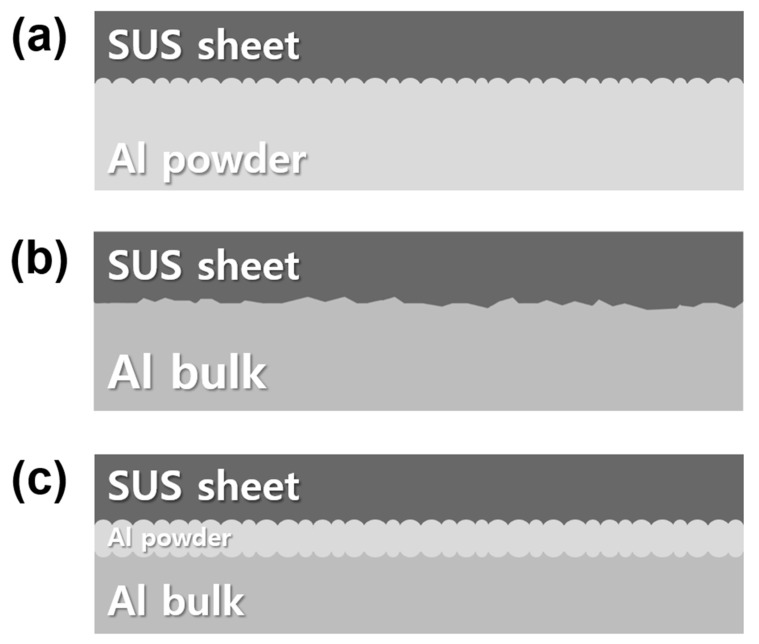
Schematic diagrams of interface layers between Al and SUS matrix: (**a**) P/B, (**b**) B/B, and (**c**) B/P/B clads.

Figure 6 shows the flexural-strength curves of the Al/SUS clads, which were determined via three-point bending tests. Figure 7 shows the schematic diagrams of the interlayers of each Al/SUS clad. In the case of the Al/SUS (P/B) and Al/Al/SUS (B/P/B) clads, flexural strength presented smooth curves because of the smooth interlayers of powder and bulk, as shown in Figure 7a,c. However, the flexural strength of the Al/SUS (B/B) clad presented a rough curve with a few kink sites, which are marked with red arrows in Figure 6. This was attributed to the coarse interlayer between Al bulk and SUS sheet, as shown in Figure 7b.

The flexural-strength values of the Al/SUS (P/B), Al/SUS (B/B), and Al/Al/SUS (B/P/B) clads were 204.0, 133.5, and 132.7 MPa, respectively. Importantly, delamination between Al and SUS was not detected in any of the Al/SUS clads after the three-point bending test. In addition, the Al/SUS (P/B) clad presented the highest flexural strength among all the specimens. This implied that powder and bulk exhibited greater strength than between bulk and bulk because of the large surface area of the powder. We expected the ICs to be brittle and negatively influence the mechanical strength of the Al/SUS clads. However, ICs could help create strong chemical bonds in metal-matrix composites [27,28]. Additionally, this strong bonding might help in effectively transferring stress in the materials [28]. Therefore, in our study, ICs with a thickness of about 20 μm were expected to form strong chemical bonds between the Al and SUS matrix, which could help transfer the load. This also suggested that SPS was suitable for producing clad composite materials with strong bonding forces between Al and SUS interfaces. On the other hand, we assumed that IC thickness significantly influences mechanical strength. In previous research, we found that the temperature of the SPS process affected IC thickness [18]. We aim to investigate the relationship between IC thickness, mechanical properties, and SPS temperature in future work.

## 4. Conclusions

Al/SUS clad materials were successfully fabricated via SPS using Al powder and bulk, and an SUS sheet. During SPS process, ICs layers of about 20 μm thickness and Vickers hardness of about 549–714 HV were created from the reaction between the Al and SUS matrices. The Al/SUS clad in the powder/bulk system showed the highest thermal conductivity of 159 W/mK and the lowest thermal expansion coefficient of 15 × 10^−6^/°C. We assumed that the interface between Al powder and SUS bulk formed an efficient heat-transfer interface that could withstand mechanical stresses caused by shrinkage and expansion. In addition, the Al/SUS (P/B) clad had the highest flexural strength of about 204 MPa. We assumed that a stronger bond was formed between powder and bulk rather than between bulk and bulk because of the large surface area of the powder. Moreover, ICs likely helped to increase the bonding strength between the Al and SUS matrices for subsequent effective stress transfer. Consequently, in the production of Al/SUS clad composite materials, the SPS process of SPS under the powder/bulk condition could very usefully be applied. The Al/SUS clad materials fabricated in this study are suitable for use in applications in various engineering fields requiring materials with high heat dissipation and high heat resistance.

## Figures and Tables

**Table 1:** Densities and thermal properties of Al/SUS clads.

Sample	Density		
	Theoretical Density (g/cm^3^)	Experimental Density (g/cm^3^)	Relative Density (%)
Al/SUS (P/B)	2.964	2.869	96.8
Al/SUS (B/B)	3.228	3.090	95.7
Al/Al/SUS (B/P/B)	3.101	2.994	96.5
